# Strength–Ductility Synergy in Biodegradable Mg-Rare Earth Alloy Processed via Multi-Directional Forging

**DOI:** 10.3390/jfb16100391

**Published:** 2025-10-18

**Authors:** Faseeulla Khan Mohammad, Uzwalkiran Rokkala, Sohail M. A. K. Mohammed, Hussain Altammar, Syed Quadir Moinuddin, Raffi Mohammed

**Affiliations:** 1 Department of Mechanical Engineering, College of Engineering, King Faisal University, Al-Ahsa 31982, Saudi Arabia; 2 Department of Mechanical Engineering, Aditya University, Surampalem 533437, India; 3 Department of Aerospace and Mechanical Engineering, The University of Texas at El Paso, El Paso, TX 79968, USA; 4 Department of Metallurgical and Materials Engineering, National Institute of Technology Andhra Pradesh, Tadepalligudem 534101, India

**Keywords:** Mg-Zn-Nd-Gd alloy, multi-directional forging, grain refinement, mechanical properties, corrosion and bioimplants

## Abstract

In this study, a biodegradable Mg-Zn-Nd-Gd alloy was processed via multi-directional forging (MDF) to evaluate its microstructural evolution, mechanical performance, and corrosion behavior. Electron backscattered diffraction (EBSD) analysis was conducted to evaluate the influence of grain size and texture on mechanical strength and corrosion resistance. The average grain size decreased significantly from 118 ± 5 μm in the homogenized state to 30 ± 10 μm after six MDF passes, primarily driven by discontinuous dynamic recrystallization (DDRX). Remarkably, this magnesium (Mg) alloy exhibited a rare synergistic enhancement in both strength and ductility, with ultimate tensile strength (UTS) increasing by ~59%, yield strength (YS) by ~90%, while elongation improved by ~44% unlike conventional severe plastic deformation (SPD) techniques that often sacrifice ductility for strength. This improvement is attributed to grain refinement, dispersion strengthening from finely distributed Mg_12_Nd and Mg_7_Zn_3_ precipitates, and texture weakening, which facilitated the activation of non-basal slip systems. Despite the mechanical improvements, electrochemical corrosion testing in Hank’s balanced salt solution (HBSS) at 37 °C revealed an increased corrosion rate from 0.1165 mm/yr in homogenized condition to 0.2499 mm/yr (after six passes of MDF. This was due to the higher fraction of low-angle grain boundaries (LAGBs), weak basal texture, and the presence of electrochemically active fine Mg_7_Zn_3_ particles. However, the corrosion rate remained within the acceptable range for bioresorbable implant applications, indicating a favorable trade-off between mechanical performance and degradation behavior. These findings demonstrate that MDF processing effectively enhances the strength–ductility synergy of Mg-rare earth alloys while maintaining a clinically acceptable degradation rate, thereby presenting a promising route for next-generation biomedical implants.

## 1. Introduction

Biodegradable implants are the new generation implants, gaining more attention due to their degradable properties without a nontoxic nature [1,2]. The major advantages of biodegradable implants are to avoid secondary surgeries, thus patients feel comfort and a reduction in hospital stay costs [3]. For a few decades, polymer-based biodegradable implants have been used for surgeries and implantations [4]. However, the primary problem with polymer-based composites fail due to their lower strength and bacterial infections. Therefore, researchers are looking for metal-based degradable implants that possess high, acceptable strength and good biological properties. The major metal degradable materials are Mg- and Iron (Fe)-based alloys [5]. However, Mg and its alloys are widely used as biodegradable metal implants due to their unique properties, such as biocompatibility and a Young’s modulus similar to natural bone, which make them more suitable than Fe-based alloys [6,7]. Various Mg-based alloys, such as Mg-Ca, Mg-Al, Mg-Zn, and Mg-rare earth element alloys, are being developed to produce high-quality metal biodegradable implants [8]. Alloys such as Mg-Y, WE43, etc., are the recent wrought alloys used in real-time clinical applications [9,10]. However, some challenges are associated with the currently available commercial Mg-based implants. One of the major limitations is low strength and early degradation [11,12].

Recent studies are focused on developing Mg-based biodegradable implants with sufficient strength, controlled degradation rates, and excellent biocompatibility. Several methods such as alloying [13,14,15], composites [16,17,18], modifying the microstructure using SPD techniques such as equal channel angular pressing [19], MDF or multi axial forging (MAF) [20], friction stir processing [21], high pressure torsion, accumulative roll bonding [22], and cyclic extrusion and compression [23] are used. Out of these processes, MDF/MAF has been adopted for processing Mg materials at different processing conditions (strain and temperature). Further, this process is found to be the most promising and unique for miniature applications such as biomedical devices, which require both high strength and acceptable corrosion resistance [24,25]. Anne et al. [26] characterized the Mg-4Zn alloy processed at 280 °C up to five passes. The results reported that the corrosion properties increased with increasing number of MDF passes. This was attributed to the grain refinement and thus an increase in length/area of grain boundaries, which acted as a physical barrier. The samples processed at five passes exhibited a very fine grain structure, and mechanical properties of the alloy increased up to three passes and reduced further, which was a result of strain hardening and inverse Hall-Petch mechanism [26]. Similar results were observed by Ramesh et al. [27] when an Mg-6Zn alloy was processed up to five passes and obtained a minimum grain size of 5 µm, possessing a large number of dislocations and high-angle grain boundaries (HAGBs). There was a significant improvement in the tensile properties and corrosion resistance up to five passes, which was attributed to the grain refinement, high dislocation densities, and strain hardening.

Similarly, Cao et al. conducted MDF of Mg-4Zn-2Gd-0.5Ca alloy at 350 °C. They observed that the corrosion resistance of the alloy varied under different processing conditions, which they attributed to the presence of second-phase particles, specifically Mg_3_Zn_6_Gd and Mg_3_Zn_3_Gd_2_. Homogenized samples exhibited better corrosion resistance compared to MDF samples. Further, with an increase in the number of MDF passes, Mg_3_Zn_3_Gd_2_ volume fraction increased, which led to greater corrosion rates [28]. Bahmani et al. [20] investigated the effect of processing temperature in MDF on XM11 Mg alloy containing 0.59 wt. % Ca and 0.52 wt. % Mn and obtained better tensile properties when processed at 300 °C, whereas better corrosion properties were found at 220–300 °C. However, an increase in the temperature led to an increase in the corrosion rate, and this was attributed to the action of second-phase particles [20].

Nevertheless, several investigations reported that the second-phase particles play a significant role in enhancing the corrosion resistance in the Mg-rare earth alloys. To improve biological properties, different rare earth elements are alloyed with Mg. Among various rare earth elements, gadolinium (Gd) and neodymium (Nd) showed good improvement in in vitro cytotoxicity behavior [29]. Studies have shown that adding a small amount of Gd and Nd (less than 2 wt. %) as an alloying element does not significantly influence biocompatibility behavior [30,31]. However, the combination of mechanical properties and corrosion resistance is a rare aspect of Mg-rare earth alloys. Indeed, the development of high-strength and corrosion-resistant biocompatible Mg alloys is quite challenging. Therefore, in the current study, the authors attempted to develop high-strength corrosion-resistant Mg alloys for bioimplant applications. For this, a Mg-rare earth alloy, i.e., Mg-Zn-Nd-Gd alloy, has been cast using the stir casting technique and later carried out the MDF till six passes. Further, the microstructural, mechanical, and corrosion properties have been studied. Finally, the effect of MDF processes on microstructures has corroborated corrosion resistance of the Mg-Zn-Nd-Gd alloy.

## 2. Materials and Methods

### 2.1. Multi-Directional Forging

The rare earth elements Gd and Nd (99.9 wt. % purity) were procured from metal industries, Mumbai, India. The Mg-Zn-Gd-Nd alloy was synthesized in a mild steel crucible under a protective atmosphere of argon. The chemical composition of the alloy is obtained from spark optical emission spectroscopy (OES), and it is given in Table 1.

**Figure 1:**
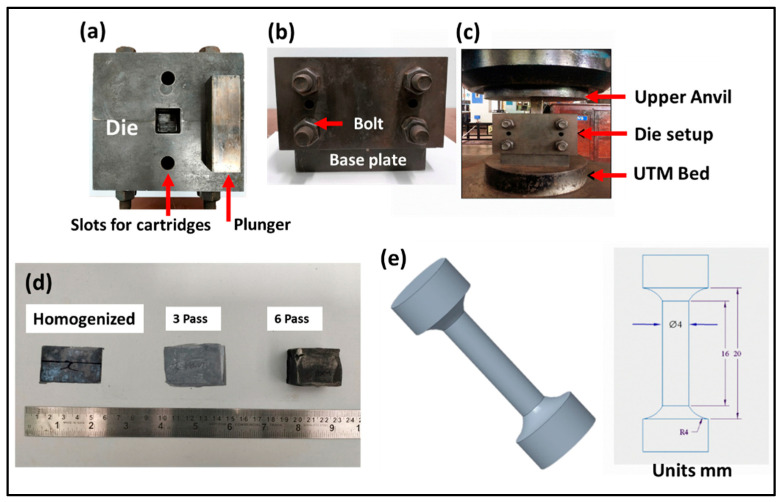
(**a**–**c**) MDF components and (**d**) MDF samples processed at different conditions, and (**e**) tensile sample dimensions.

The complete experimental setup details of the MDF are shown in Figure 1a–c. As-received blocks were homogenized as per ASTM B 661 (Standard Practice for Heat Treatment of Magnesium Alloys. ASTM International: West Conshohocken, PA, USA, 2019) at 400 °C for 24 h, followed by air cooling to room temperature. The homogenized Mg-Zn-Gd-Nd alloy blocks were sectioned into small rectangular samples of dimension 30 mm × 30 mm × 20 mm obtained through wire-cut EDM machining. The samples were forged at a uniform strain rate of 2 mm/min at 250 °C. After each pass of MDF, the samples were rotated to their next forging axis, and the same was repeated for the whole cycle. Figure 1d shows the samples processed after MDF under different conditions.

### 2.2. Microstructural Characterization

The samples for all the microstructure characterizations were extracted from the plane parallel to the final forging axis. The samples were sequentially polished by SiC abrasive papers with different granularities from 120 to 2000 grit size, followed by mechanical buffing using a velvet polishing cloth. To eliminate the scratches induced by mechanical grinding and to prevent the rise in temperature in this process, diamond suspension with a particle size of 1–3 μm, along with kerosene, was also utilized during elaborate polishing.

The samples were etched with a solution containing 10 g oxalic acid + 1 mL nitric acid + 4 mL acetic acid + 200 mL distilled water for 10–20 s. The surface morphology of homogenized, 3-pass, and 6-pass samples was observed using an Optical Microscope (OM, Zeiss, Aixolab A1, Jena, Germany) and Scanning Electron Microscope (SEM, Carl Zesis, Jena, Germany) equipped with EDX. The samples for EBSD were prepared by sequential polishing using SiC abrasive papers from 120 to 2000 grit, followed by electropolishing at 16 V for 60 sec at −25 °C using Struers AC2 electrolyte as per ASTM E1558-09(Standard Guide for Electrolytic Polishing of Metallographic Specimens. ASTM International: West Conshohocken, PA, USA, 2009). The EBSD results were analyzed using the HKL Channel 5 software. X-ray diffraction (XRD) analysis on the homogenized and MDF samples was performed using Empyrean 3rd Gen, Malvern PANalytical diffractometer (Almelo, Netherlands) within the range of 10–110° using Cu-Kα source having a wavelength of 1.54 Å at a scan rate of 5 °/min. The linear intercept method has been employed to measure the grain size.

### 2.3. Mechanical Properties

Tensile samples were extracted from the blocks of the homogenized and MDF samples as per ASTM E8 standards (Standard Test Methods for Tension Testing of Metallic Materials. ASTM International: West Conshohocken, PA, USA, 2022) with a gauge length of 16 mm. The dimensions of the tensile samples are given in Figure 1e. Tensile testing of 3 samples from each condition (homogenized, 3 pass, and 6 pass) was performed on the universal testing machine (Shimazdu AG-X plus^TM^, 100 KN, Kyoto, Japan), at a cross-head speed of 1 mm/min at room temperature. Here, the tensile cross-head is parallel to the forging axis. The fracture surface morphology of the ruptured samples was analyzed through SEM. Microhardness test of the homogenized and MDF samples was performed as per ASTM-E384 Standards (Standard Test Method for Microindentation Hardness of Materials. ASTM International: West Conshohocken, PA, USA, 2023) on the Omni Tech Microhardness testing machine. The samples were subjected to a load of 100 gf, and a dwell time of 10 s was employed to calculate the average microhardness for all the samples.

### 2.4. Electrochemical Corrosion Testing

The electrochemical corrosion studies of the homogenized and MDF samples of dimensions 10 mm × 10 mm × 4 mm in Hank’s body solution at 37 ± 0.1 °C were investigated using a traditional three-electrode system in an electrochemical workstation (OrigaFlex chemical workstation). The three-electrode system had a saturated calomel electrode (SCE) as the reference electrode, platinum wire as the counter electrode, and the sample as the working electrode, followed by which the whole three-electrode setup was placed in a water bath at a temperature of 37 ± 0.1 °C. Prior to measuring the open circuit potential (OCP), the samples were immersed for about 5 min in Hank’s solution to stabilize potential, and then OCP was measured for about 30 min from −100 to 1000 mV. Electrochemical Impedance Spectroscopy (EIS) measurements were made with a scan rate of 20 mV/sec at the frequency range of 100 KHz to 10 mHz. Potentiodynamic polarization (PDP) tests were then carried out with a scan rate of 1 mV/s after the EIS measurements. From the current density-potential data, Tafel plots were generated. Corrosion rate (mm/yr) was calculated according to ASTM G102 (Standard Practice for Calculation of Corrosion Rates and Related Information from Electrochemical Measurements. ASTM International: West Conshohocken, PA, USA, 2015). The corrosion rate was quantified using the following Equation (1) [32] given below.

(1) $$P_{i}=22.85\;I_{\mathrm{Corr}}$$

where $P_{i}$ is corrosion rate in mm/yr and I_corr_ in mA/cm^2^.

## 3. Results and Discussion

### 3.1. Microstructure and Phase Evolution of the MDF Samples

**Figure 2:**
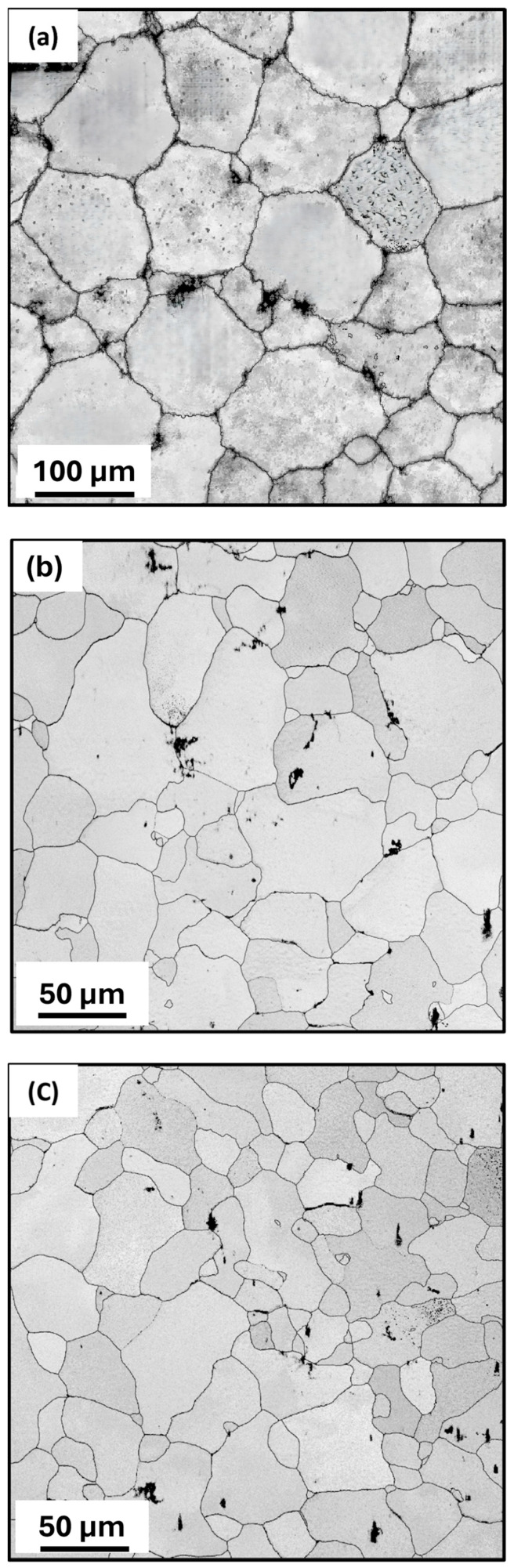
Optical Microstructures: (**a**) homogenized, (**b**) three-pass, and (**c**) six-pass samples.

**Figure 3:**
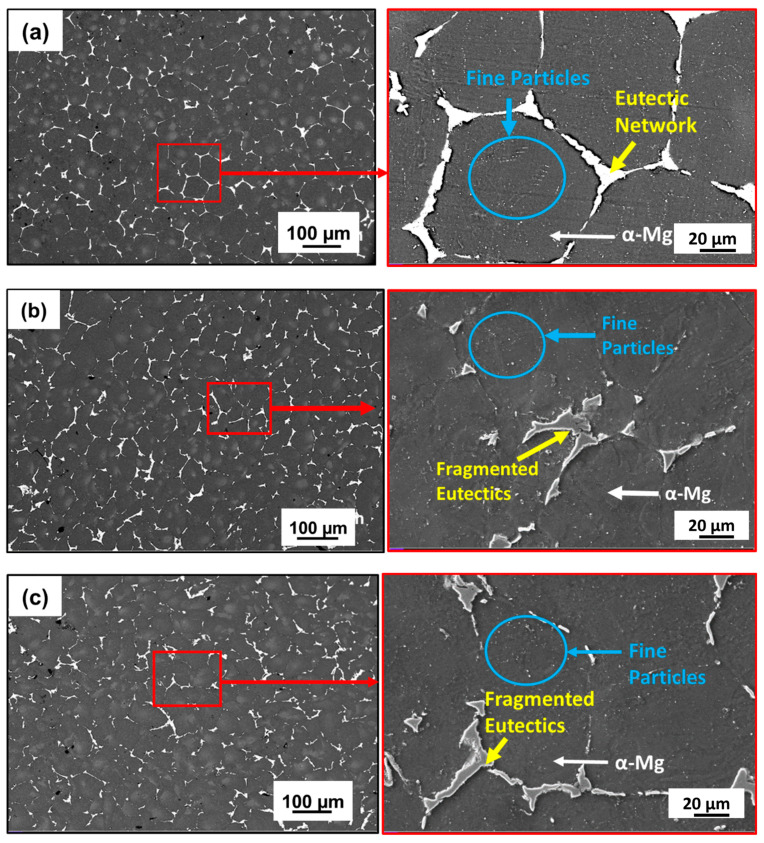
SEM Microstructures: (**a**) homogenized (continuous eutectic network), (**b**) three-pass (semi-continuous eutectic network), and (**c**) six-pass samples (discontinuous eutectic network).

The OM and SEM microstructures of the homogenized and MDF (three-pass and six-pass) samples are shown in Figure 2 and Figure 3, respectively. The as-cast sample was homogenized at 400 °C for 24 h to obtain uniformity and to dissolve micro irregularities formed during casting and alloying. The homogenized sample exhibited equiaxed honeycomb-shaped grain structure (α-Mg) as shown in Figure 2a with an average grain size of 118 µm ± 5 µm. Further, the grain boundaries were clearly visible and decorated with a continuous eutectic network of second-phase particles along the grain boundaries and fine particles in the grain interior. This can be clear from the SEM microstructures given in Figure 3a. After three passes of MDF, the microstructure exhibited a mixture of coarse and fine grains distributed in an uneven manner (Figure 2b) with an average grain size of 40 ± 10 µm.

Similar observations in the MDF-processed Mg-rare earth alloys were reported by a few researchers [33,34]. The obtained bimodal grain structure formed during MDF at a cumulative strain of ε_3_ = 1.2, driven by DDRX. This strain regime promoted partial recrystallization, resulting in a mix of fine DRXed grains (~1–5 µm) and residual coarse grains (~20–110 µm). After six passes of MDF, the grain size was further reduced up to 30 ± 10 µm due to subdivision of grains and the formation of new fine grains inside the grain. It has been observed that with an increase in the cumulative strain (ε_6_ = 2.43), grain size decreases. The eutectic network of second-phase particles observed along the grain boundary in the homogenized condition is fragmented during the MDF process, dispersed, and distributed non-uniformly throughout the α-Mg matrix. This can be confirmed from the SEM microstructures provided in Figure 3b,c. These second-phase particles will enhance the DDRX through particle-stimulated nucleation (PSN), leading to formation of fine grains along the grain boundaries and inside the grains [35]. Finally, the microstructure turns into a bimodal grain microstructure, as seen in Figure 2c.

**Figure 4:**
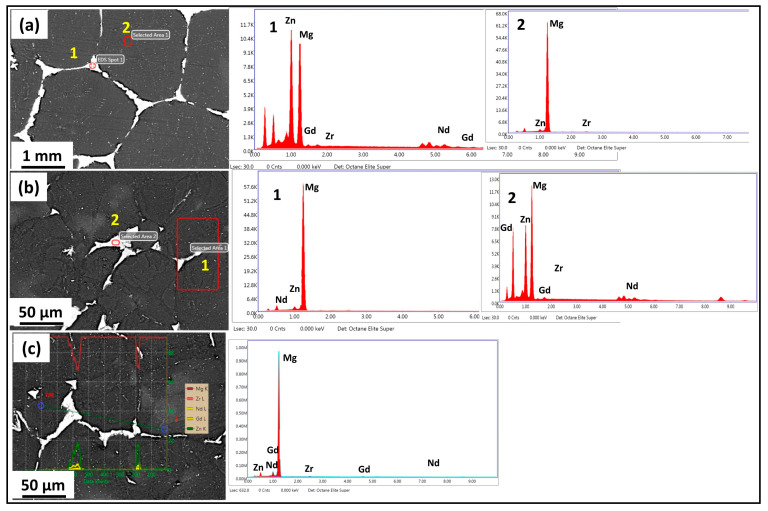
EDS Mapping: (**a**) homogenized, (**b**) 3-pass, and (**c**) 6-pass samples.

Figure 4 shows the EDS mapping of the homogenized and MDF samples. A higher percentage of α-Mg-rich phases and Zn-Nd-rich secondary phases were present in the microstructure of the Mg-Zn-Gd-Nd alloy, which was supported by the EDS mapping shown in Figure 4a, which revealed a distinct atomic contrast for the elements Mg, Zn, and Nd. The eutectic network of second-phase particles in the homogenized sample had a continuous type of distribution along the grain boundaries, as shown in Figure 3a and Figure 4a. After three passes of MDF, the second-phase particle distribution was found to be semi-continuous in a uniform manner along the grain boundaries. The presence of very fine second-phase particles near and inside the grain boundaries (Figure 3b and Figure 4b) has also been observed. Further, after six passes of MDF, the second-phase particle distribution was discontinuous along the grain boundaries and was distributed in a non-uniform manner (Figure 3c and Figure 4c). The density of fine second-phase particles was increased compared to three passes, and the same can be reflected in Figure 3c and Figure 4c. This is mainly due to high cumulative strain during the MDF process and leads to the formation of strain-induced precipitation, and similar observations were reported by Cao et al. [28] and Yang et al. [36].

**Figure 5:**
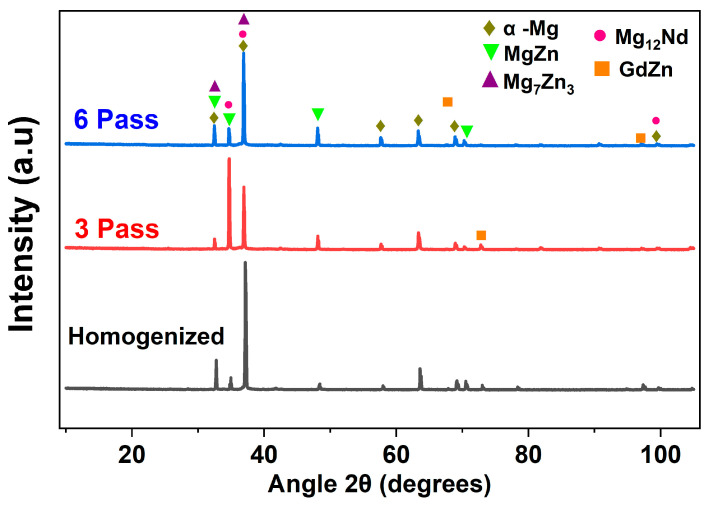
XRD patterns of the homogenized and MDF samples.

Figure 5 shows the XRD patterns of the homogenized and MDF samples. For all three conditions, α-Mg was exhibited as the major phase; however, after the MDF passes, the peaks corresponded to different second-phase particles such as MgZn, Mg_7_Zn_3_, and Mg_12_Nd. The variation in the intensity of the peaks corresponding to Mg_12_Nd and Mg_7_Zn_3_ at different processed conditions can be observed due to the dissolution of particles; further, the major second-phase particles of Mg-Zn-Gd-Nd alloy can be considered as Mg_12_Nd and Mg_7_Zn_3_, as observed in XRD and the EDX composition; these observations were consistent with the authors’ report in [30,37]. Hence, through XRD phase analysis, it is confirmed that the eutectic network is Mg_12_Nd, and fine second-phase particles are Mg_7_Zn_3_. Further, no peaks corresponded to the GdZn phase in the six-pass samples due to the dissolution of particles.

**Figure 6:**
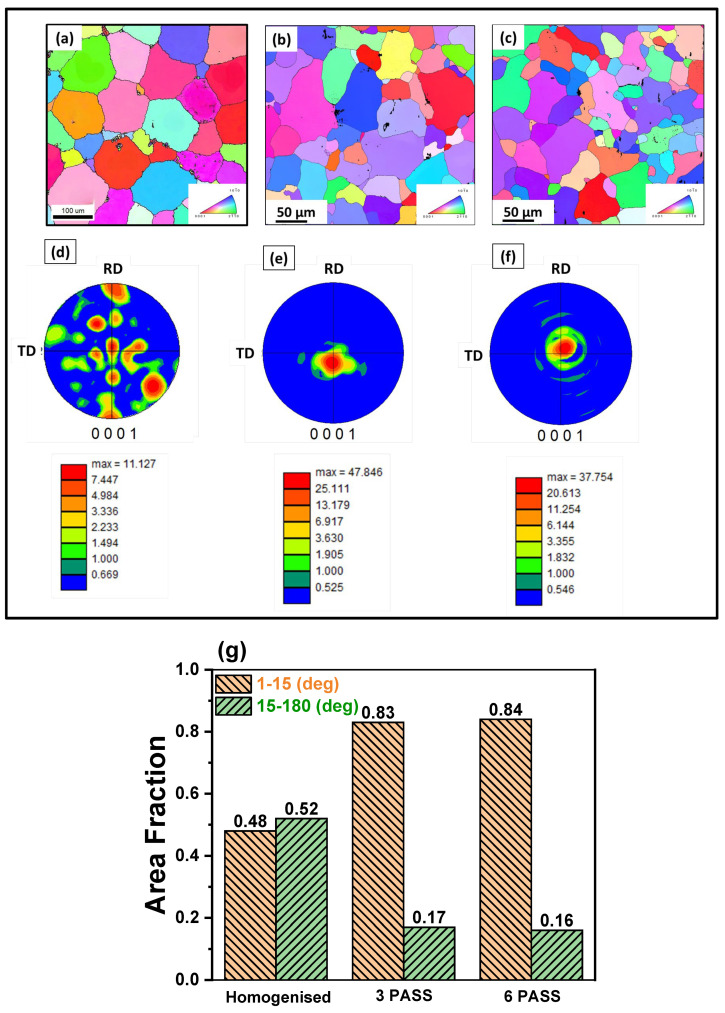
Inverse pole figures: (**a**) homogenized, (**b**) 3-pass, and (**c**) 6-pass samples; pole figures: (**d**) homogenized, (**e**) 3-pass, (**f**) 6-pass samples, and (**g**) grain boundary misorientation distribution.

Inverse pole figure (IPF) and pole figure (PF) maps were obtained through EBSD analysis of the samples processed at different conditions indicated in Figure 6. It was observed that the homogenized sample exhibited an equiaxed honeycomb-type grain structure with an average grain size of 118 ± 5 µm (Figure 6a). Upon MDF processing, a mixture of both fine and coarse grains, i.e., a bimodal grain structure, was observed in both the three-pass and six-pass samples, and further, no traces of twins were generated (Figure 6b,c). This can be attributed mainly to three possible reasons: (i) the high temperature during MDF, (ii) the orientation of grains during deformation that restricted the twinning activation, and (iii) reduction in grain size (for three pass 40 ± 10 µm and for six pass 30 ± 10 µm) upon increase in strain accumulation with increase in MDF passes. However, a significant reduction in grain size was observed after the MDF passes. Primarily, a random texture (Figure 6d) was observed in the homogenized sample. After MDF, the random texture turned into a near-net strong basal texture. The basal (0001) PFs (Figure 6e,f) confirm the presence of texture but are slightly weakened along the basal plane (0002) after the MDF passes. The presence of random texture in the Mg-rare earth alloys after SPD is quite common and widely reported [38,39,40,41].

The distribution of grain boundary misorientation angles is presented in Figure 6g. A marked increase in the fraction of LAGBs (1–15°) is observed in the MDF-processed samples, rising from 48% in the homogenized condition to 84% post-processing. This increase is primarily attributed to the formation of fine sub-grains and a high density of dislocations generated during the MDF process. Concurrently, a significant reduction in the fraction of HAGBs (15–180°) is observed, decreasing from 52% in the homogenized sample to 17% in the MDF-processed samples. Notably, the maximum fraction of HAGBs was found in the homogenized condition. The higher fraction of HAGBs in the homogenized sample is known to enhance corrosion resistance in Mg-rare earth alloys [38], a point that will be further discussed in Section 3.3 on corrosion behavior.

### 3.2. Mechanical Response

#### 3.2.1. Tensile Behavior and Fracture Analysis

**Figure 7:**
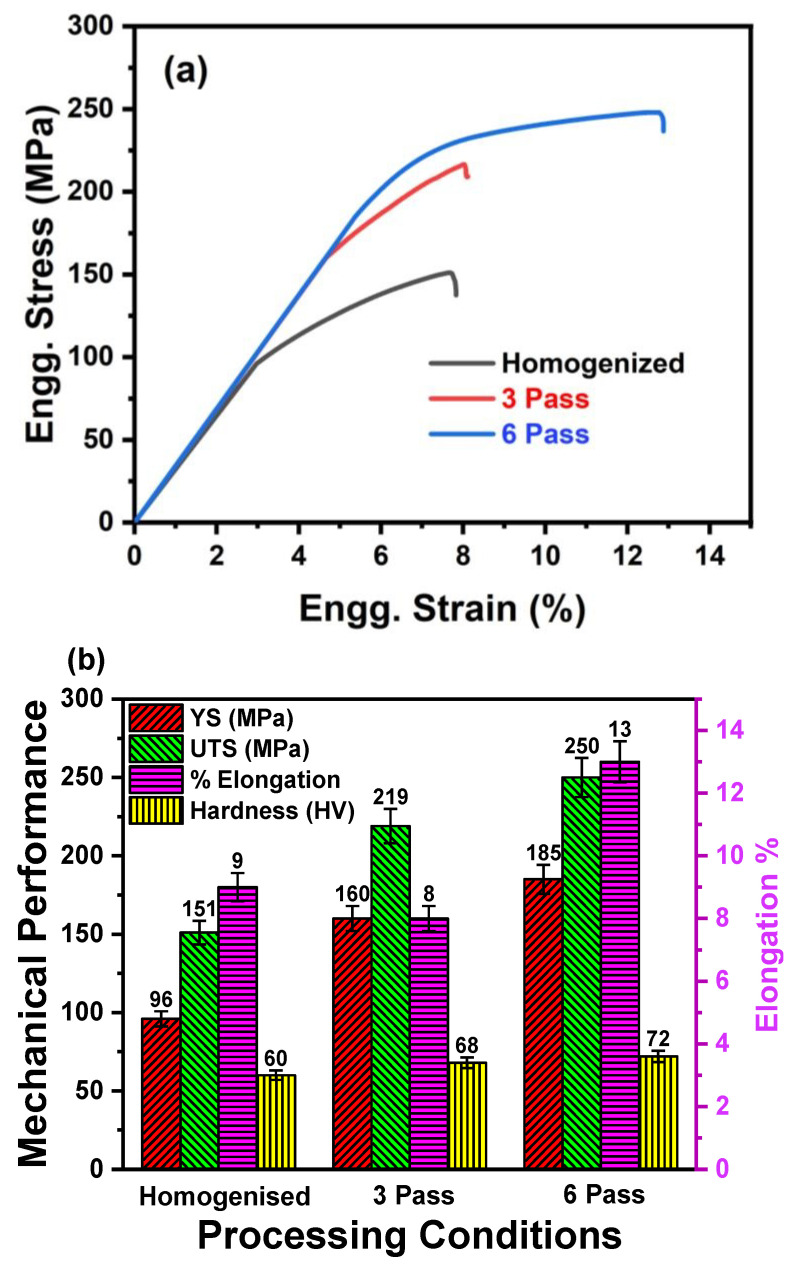
(**a**) Engineering stress–strain curve of the samples processed at different conditions, and (**b**) comparison of UTS and elongation % of the samples processed at different conditions.

Engineering stress–strain curves of the homogenized and MDF samples are plotted in Figure 7a. Figure 7b summarizes the mechanical properties of samples processed at different conditions. It was observed that the YS, UTS, and % of elongation were increased with increasing number of MDF passes. The YS and UTS of the homogenized sample were 96 ± 3 MPa and 151 ± 4 MPa, respectively. The maximum UTS was observed in the six-pass sample (250 ± 7 MPa), followed by three-pass (219 ± 6 MPa), and then homogenized (151 ± 4 MPa). A similar trend was observed in the YS and % elongation of the six-pass sample. Indeed, it was noticed that overall, a 59% increase in UTS, 92% increase in YS, and 40% increase in elongation were observed in the six-pass sample compared to the homogenized sample. This unique combination of highest strength and ductility was significant and very special for bioimplant applications [42]. The increase in the YS and UTS after MDF can be primarily attributed to MDF-induced grain refinement due to high strain accumulation and dispersion strengthening from second-phase particles Mg_12_Nd and Mg_7_Zn_3_ [43,44]. After six passes of MDF, the continuous eutectic network had fragmented into discontinuous particles and distributed non-uniformly in the α-Mg matrix. This could restrict the premature failure of the sample and enhance the ductility. Further, the significant enhancement in the elongation of the three- and six-pass samples is primarily attributed to the weak basal texture observed in Figure 6e,f obtained after the MDF process. The weakening of texture in Mg-rare earth alloys helps in enhancing the ductility of samples. This phenomenon is well addressed and documented by the researchers and authors’ previous findings [39,40]. In addition, the MDF samples exhibited outstanding elongation because activation of different slip systems that are introduced after texture randomization really boosted the elongation [45,46].

#### 3.2.2. Microhardness Evolution

The microhardness results of the samples processed under different conditions are presented in Figure 7b. The homogenized sample exhibited an average microhardness of 60 ± 1 HV, which was the lowest among all the conditions. A notable increase in microhardness was observed after MDF of the Mg-Zn-Nd-Gd alloy. Specifically, the samples processed through three and six passes exhibited average microhardness values of 68 ± 1.2 HV and 72 ± 1.7 HV, respectively. However, the increase from three to six passes was relatively modest, which can be attributed to the complete dissolution of the GdZn phase after six passes, as confirmed by the XRD analysis (Figure 5). Overall, the MDF process led to an enhancement of approximately 20% in microhardness compared to the homogenized condition. This improvement can be primarily ascribed to grain refinement (Figure 6), strain hardening, and dispersion strengthening resulting from the presence of secondary phase particles such as Mg_12_Nd and Mg_7_Zn_3_ along both the grain boundaries and interiors of the MDF-processed samples [47].

**Figure 8:**
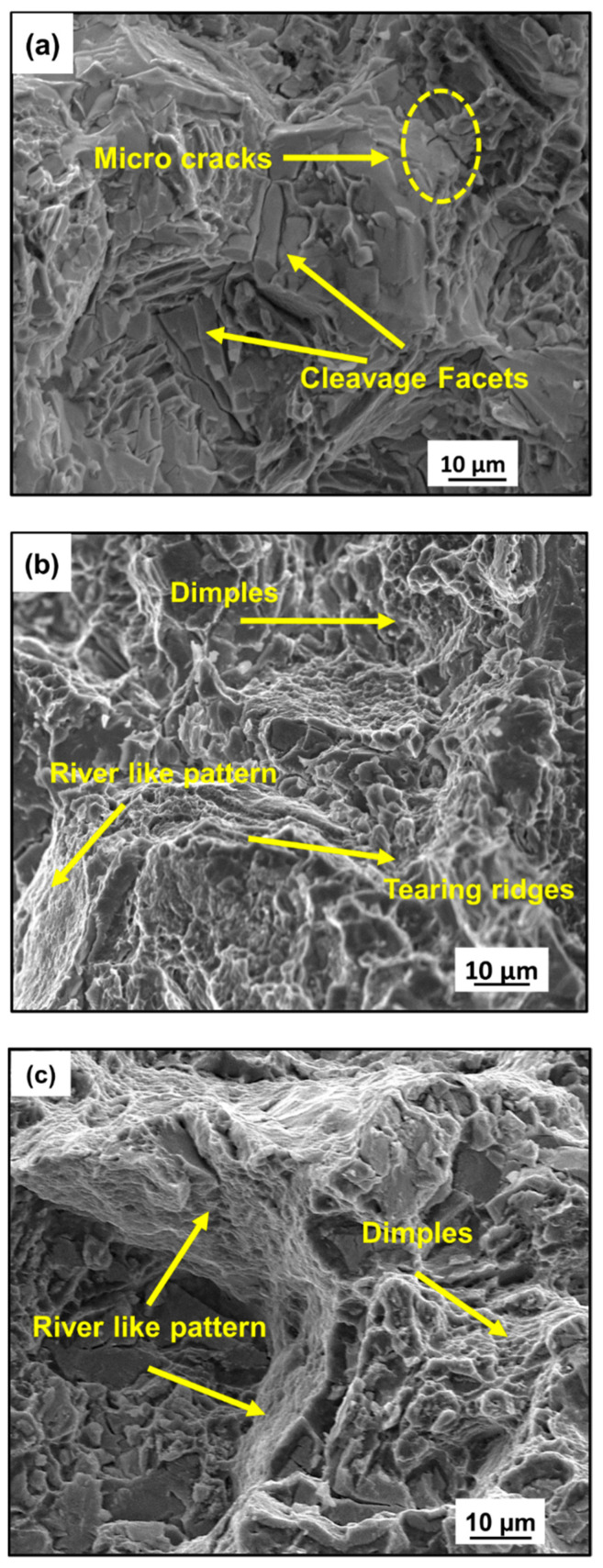
Fractography images: (**a**) homogenized, (**b**) 3-pass, and (**c**) 6-pass samples.

Figure 8 reveals the fracture surface of the homogenized and MDF samples. The homogenized sample fracture surfaces revealed tearing ridges and micro cracks leading to macro-ones (Figure 8a). The presence of a tearing edge and cleavage facets is an indication of brittle fracture. The convergence of certain micro cracks to macro crack, leading to the formation of a tearing edge, has been observed (Figure 8a). Mg alloys usually have a brittle type of failure due to the restricted dislocation system; the formation of the cleavage facets can be attributed to the presence of large α-Mg grains [48]. However, the sample processed for three passes exhibited the presence of dimples of different dimensions and also a river-like pattern, as shown in Figure 8b. This indicates that the three-pass sample showed a mixed mode of fracture (brittle + ductile) with a considerable percentage of elongation. This can be attributed to the grain refinement because of MDF processing. Reduction in tearing edges and the size of dimples was observed in the sample processed up to six passes, as shown in Figure 8c. Compared to the three-pass and six-pass samples, it had a greater grain refinement in microstructure; as a result, the reduction in dimple size and increase in elongation percentage could be observed. Fracture of the samples processed at six passes is a combination of brittle and ductile fracture, and is more dominated by a ductile nature. The depth of the dimple depends on the microstructure and plasticity of the material [49,50].

### 3.3. Electrochemical Corrosion Behavior

#### 3.3.1. Potentiodynamic Polarization (PDP) Studies

**Figure 9:**
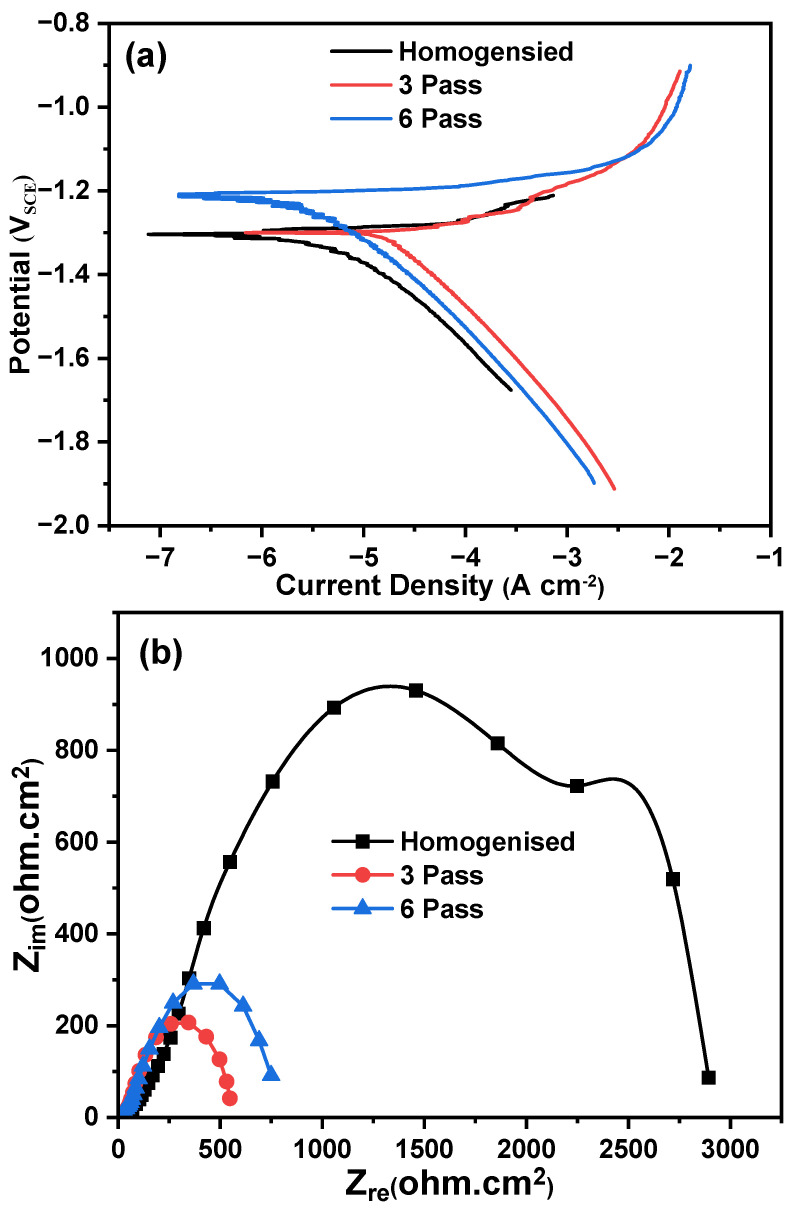

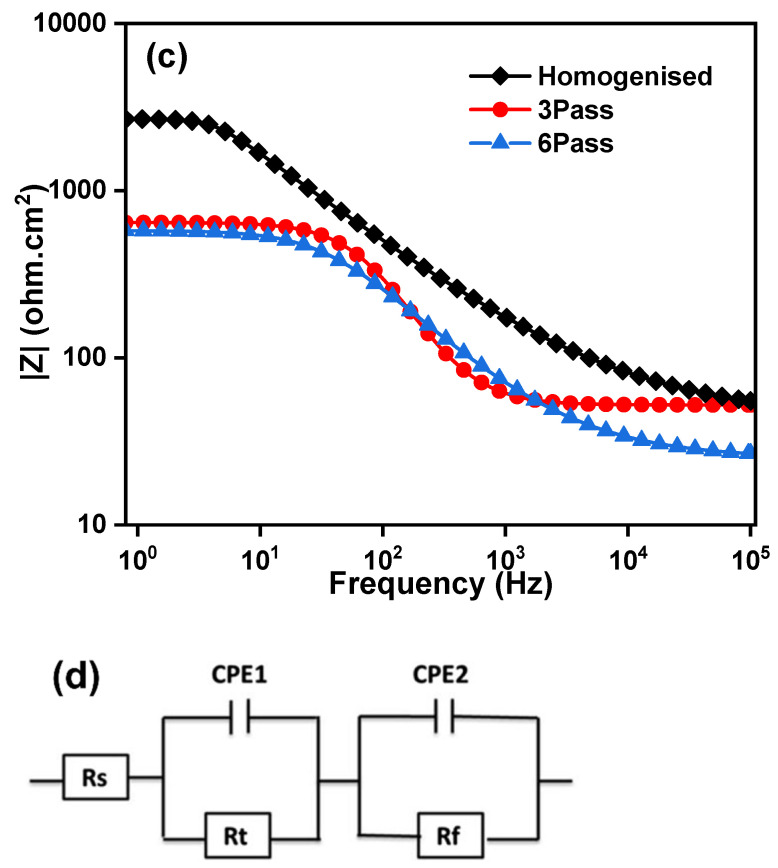
(**a**) PDP curves, (**b**) Nyquist plot, (**c**) Bode plots of samples processed at different conditions, and (**d**) equivalent EIS circuit.

PDP curves of the homogenized and MDF samples tested at 37 ± 1 °C in Hank’s body solution are plotted in Figure 9a. The measured parameters, such as corrosion potential (E_corr_), corrosion current density (I_corr_), anodic slope (βa), and cathodic slope (βc), were calculated from Tafel plots and are tabulated in Table 2. From Table 2, it can be inferred that the corrosion rate increased after the MDF process. However, the corrosion current density is directly proportional to the corrosion density; with an increase in corrosion current, the corrosion rate also increases. The cathodic curve represents the evolution of hydrogen when subjected to corrosion, whereas the anodic curve represents the dissolution of Mg. All the samples have similar polarization curve trends, having a steep increase in anode curves due to the negative difference effect. The PDP results reveal that the homogenized sample exhibits the most noble corrosion potential (−1.2799 V) and the lowest corrosion current density (0.0051 mA cm^−2^), while the three-pass MDF specimen shows the highest corrosion current density (0.0199 mA cm^−2^) and the least charge-transfer resistance (34.9 ohm·cm^2^) derived from the EIS analysis. The six-pass sample shows an intermediate behavior (I_corr_ = 0.0109 mA cm^−2^; *R_t_* = 144.5 Ω·cm^2^). These variations are consistent with the microstructural evolution during MDF, which modifies grain-boundary character, texture, and intermetallic distribution. Table 2 shows that the homogenized sample had a lower corrosion rate of 0.1165 mm/yr compared to the three- and six-pass samples. From Figure 9a, it is observed that the homogenized sample exhibited the filiform type of corrosion, and a small part is affected by pitting corrosion. The area fraction of HAGBs is maximum (55%) for the homogenized sample. A passive layer is formed due to the high fraction of HAGBs, which could enhance the corrosion resistance for the homogenized sample [51]. After three and six passes of MDF, corrosion potential shifted to −1.2967 and −1.1999, respectively, which is decreased slightly with an increase in corrosion current density compared to the homogenized sample, hence having a slightly higher corrosion rate of 0.4560 mm/yr and 0.2499 mm/yr for three and six passes, respectively. In strongly textured Mg alloys, Song et al. [51] noticed that surfaces that had higher {0002} basal planes exhibited more corrosion resistance than those with lesser basal planes. In addition, Xin et al. [52] had investigated the effect of texture on corrosion behavior of AZ31 Mg alloy, and observed similar results, which were noticed by Song et al. Here, the MDF samples exhibited near-net strong basal texture (weak texture), and the presence of high discontinuous distribution of LAGBs could lead to galvanic corrosion attack at the grain boundaries. Grain boundaries and LAGBs exhibit more active sites and energy compared to the substrate, which acts as a highly active site. Consequently, grain refining enhances the area of grain boundaries and LAGBs, resulting in an enhanced dissolution rate of atoms at the grain boundaries and augmenting the chemical reactivity of the alloy [53]. In addition, Cui et al. [54] studied the effect of grain size and second-phase particles on the corrosion behavior of Mg-3Al-5Pb-1Ga-Y alloy. They found that the presence of a high fraction of LAGBs and fine second-phase particles enhanced the corrosion rate. After three passes, the bimodal grain structure (coarse unrecrystallized + fine DRXed grains) introduced moderate surface energy heterogeneity, with corrosion initiating preferentially at fine-grained regions (higher energy). The further refined microstructure increased the total grain boundary area and surface free energy after six passes, leading to a more pronounced corrosion rate. The presence of high fraction of grain boundary is further confirmed with the grain boundary distribution presented in Figure 6g. The weakened texture also reduced the fraction of corrosion-resistant basal planes, exacerbating material degradation [55].

#### 3.3.2. Electrochemical Impedance Spectroscopy

Nyquist and Bode plots of the homogenized and MDF samples in Hank’s body solution at 37 ± 0.1 °C are shown in Figure 9b,c. EIS provides the inductive and capacitance loop and is also used to investigate the electrochemical interface between the electrode and electrolytes. The Nyquist plot depicts a semicircle, which indicates the capacitance arc and the formation of protective film and degradation of it. The capacitance arc’s diameter represents the corrosion resistance of Mg-Zn-Gd-Nd alloy. From Figure 9b, it can be observed that all the samples exhibited depressed semicircles; also, the homogenized sample had a larger semicircle, followed by the six- and three-pass samples, indicating better corrosion resistance for the homogenized sample. The equivalent circuit used to analyze the Nyquist is shown in Figure 9c. High frequency capacitive loop of the electrode reaction process was described using a parallel circuit of electric double-layer capacitance cell (C_dl_) and charge transfer (*R_t_*). Constant phase elements (CPE1 and CPE2) and a film resistance R_f_ were introduced to the circuit. If the metals exhibit better corrosion resistance, it is difficult to transfer charges between the solution and the sample surface, so *R_t_* becomes higher. It is noticed that, *R_t_* values for the homogenized, three-pass, and six-pass samples are 244.5 ohm·cm^2^, 34.9 ohm·cm^2^, and 144.5 ohm·cm^2^, respectively. Therefore, the corrosion resistance for the homogenized sample increased when compared to the MDF samples.

**Figure 10:**
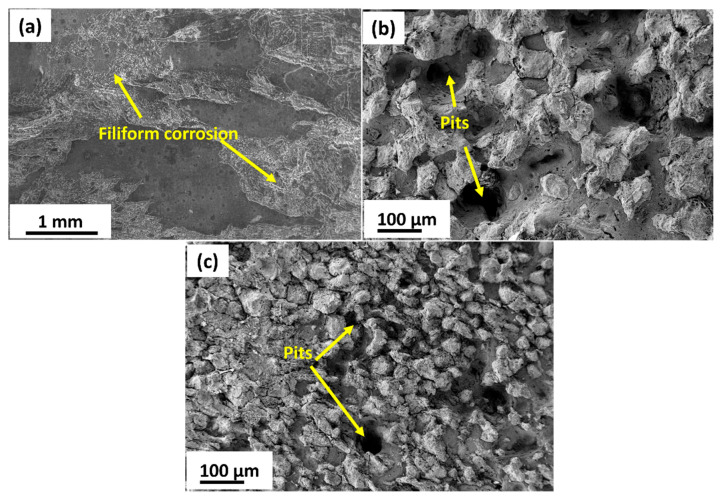
Surface morphologies after corrosion testing: (**a**) homogenized, (**b**) 3-pass, and (**c**) 6-pass samples.

Micro-galvanic coupling between the α-Mg matrix and intermetallic phases (Mg_12_Nd and Mg_7_Zn_3_) plays a decisive role. The homogenized alloy shows relatively coarse and semi-continuous Mg_12_Nd at grain boundaries, while MDF processing refines and redistributes these particles. The fine, discontinuous precipitates formed after three passes provide numerous cathodic sites adjacent to the α-Mg matrix, enhancing micro-galvanic interactions and accelerating localized dissolution. Consequently, the three-pass specimen records a higher I_corr_ and lower *R_t_*. Such particle-induced galvanic effects have been widely reported in Mg–Nd and Mg–Zn alloys, where the electrochemical potential difference between intermetallics and the Mg matrix determines local corrosion kinetics [56,57]. The corrosion rate of the MDF-processed samples up to three passes has declined compared to the homogenized sample. However, there is a slight decrease in corrosion rate of six-pass samples compared to three-pass samples, and the primary reason can be attributed to the type, phase, and distribution of the second-phase particles present in the microstructure. However, the corrosion resistance depends on the electrode potential of the matrix and the adjoining phases. The majority of the second-phase particles identified through EDX and XRD is Mg_12_Nd and Mg_7_Zn_3_ (Figure 4b,c) and Figure 5; these phases will corrode first if the potential of the phase is lower than that of the substrate. From Figure 10, it is noticed that the three- and six-pass samples have experienced pitting corrosion. The corrosion potential of second-phase particles is lower than the Mg substrate, so that the second-phase particles acted as an anode and dissolved preferentially [58]. However, as reported in the microstructure results, the distribution of the second-phase particles Mg_12_Nd was continuous among the grain boundaries in the homogenized sample, whereas semi-continuous with respect to the three- and six-pass samples. Therefore, it cannot provide better protection to the substrate if its distribution is discontinuous. The corrosion resistance of Mg alloy is closely related to the length of the grain boundary, which acts as a physical barrier [59]. The fragmented fine second-phase particles act as a cathode near the LAGBs, and the α-Mg matrix acts as an anode, which forms a micro galvanic couple. From Figure 10b,c, it is observed that pitting corrosion occurred at the grain boundaries region for both samples due to the severe galvanic effect, which could increase the corrosion rate after MDF on Mg-Zn-Nd-Gd alloy.

## 4. Conclusions

The biodegradable homogenized Mg-Zn-Gd-Nd alloy was successfully processed through MDF up to six passes at 250 °C with a cumulative strain of 2.43. Mechanical and microstructural characterizations were carried out to study the effect of MDF on the Mg-Zn-Gd-Nd alloy. To evaluate its suitability for biomedical applications, the corrosion behavior of the alloy was analyzed in Hank’s body solution at 37 °C under various processing conditions. Key findings from the study are summarized below:

Grain size reduced from 118 ± 5 µm in the homogenized condition to 30 ± 10 µm after six passes with uniform distribution of Mg_7_Zn_3_ fine particles and semi-continuous Mg_12_Nd eutectic network.

The microhardness of the six-pass MDF sample increased by 20%, while UTS and YS exhibited significant enhancements of 59% and 90%, respectively, compared to the homogenized sample. This is due to the fact that the grain refinement, strain hardening, and dispersion strengthening from the second-phase particles.

Additionally, the ductility of the six-pass MDF sample improved remarkably, with a 44% increase in elongation, attributed to texture weakening and uniform second-phase distribution (resisting premature failure).

The homogenized sample exhibited better corrosion resistance compared to MDF-processed samples. Although the corrosion rate of the six-pass MDF-processed sample (0.2499 mm/yr) is higher than that of the homogenized samples (0.1165 mm/yr), this remains within an acceptable range for biodegradable implant applications.

The slight reduction in corrosion resistance of the MDF-processed samples can be attributed to a high fraction of LAGBs and fine second-phase particles; however, it does not compromise the alloy’s suitability for biomedical use. Instead, the controlled degradation rate aligns well with the clinical requirements for biodegradable implants, where gradual material dissolution is essential for tissue regeneration.

## Figures and Tables

**Table 1:** Chemical Composition of Mg-Zn-Gd-Nd Alloy.

Elements	Zn	Nd	Gd	Zr	Mg
Composition (wt. %)	3.07	1.70	1.40	0.95	Balance

**Table 2:** Corrosion Properties of The Samples Processed at Different Conditions.

Sample Condition	I_corr_ (mA/cm^2^)	E_corr_ (V)	βa	βc	CR (mm/yr)
Homogenized	0.0051	−1.2799	0.0071	−0.1987	0.1165
3 Pass	0.0199	−1.2967	0.0285	−0.2557	0.4560
6 Pass	0.0109	−1.1999	0.0044	−0.2606	0.2499

## Data Availability

The original contributions presented in the study are included in the article, further inquiries can be directed to the corresponding author.
